# Validation of a new study skills scale to provide an explanation for depressive symptoms among medical students

**DOI:** 10.1371/journal.pone.0199037

**Published:** 2018-06-25

**Authors:** Eiad AlFaris, Farhana Irfan, Shuaa AlSayyari, Waad AlDahlawi, Shahad Almuhaideb, Alanood Almehaidib, Shaikha Almoqati, Abdullah M. A. Ahmed, Gominda Ponnamperuma, Muhannad AlMughthim, Shaik Shaffi Ahamed, Nassr Al Maflehi, Cees van der Vleuten

**Affiliations:** 1 King Saud University Chair for Medical Education Research and Development, Department of Family and Community Medicine, College of Medicine, King Saud University, Riyadh, Saudi Arabia; 2 College of Medicine, King Saud University, Riyadh, Saudi Arabia; 3 Centre for Medical Education, National University of Singapore, Singapore, Singapore; 4 Department of Family and Community Medicine, College of Medicine, King Saud University, Riyadh, Saudi Arabia; 5 College of Dentistry, King Saud University, Riyadh, Saudi Arabia; 6 Department of Educational Development and Research Maastricht University, Maastricht, the Netherlands; Universita degli Studi di Udine, ITALY

## Abstract

**Background:**

Medical students are faced with enormous academic demands that may influence their emotional wellbeing. The high rate of depression among medical students and its negative impact is an impetus to find explanation for the factors associated with it. Study skills that students possess might be such a factor. The current tools for the assessment of the study skills may have certain limitations, particularly for different cultural settings.

**Objectives:**

This study aimed to develop and validate a Study Skills Inventory (SSI), and to investigate the relationship between the students’ study skills and the extent (severity) of depressive symptoms, measured using the validated tool.

**Method:**

The first version of the SSI was developed through expert consensus. The inventory was then administered to a randomly selected group of medical students. Confirmatory factor analysis was conducted for the internal validity. External validation was conducted by comparing the results of the SSI with the “Approaches and Study Skills Inventory for Students” (ASSIST). After validation, the correlation between the SSI total score with the Beck Depression Inventory II (BDI-II) total score was investigated using the Pearson correlation coefficient. The means of the total study skills scores for each severity category of depression were compared using ANOVA.

**Results:**

A total of 23 items, representing five sub-scales, were included in the inventory. Based on 372 student responses (response rate of 93%), the five-factor solution explained a cumulative variance of 52% and Cronach alpha was 0.84. The SSI total score had a significant negative association with the BDI-II depression score (Pearson correlation of -.348** and P<0.0001).

**Conclusion:**

This study showed evidence for acceptable reliability and validity of the newly developed SSI. Poor study skills were found to correlate with higher depressive symptoms. This association needs confirmation in future research and could open a new door for better understanding of student depression.

## Introduction

Depression is a common mental health issue that affects a significant proportion of medical students [1–5]. It is a multi-faceted disorder that disrupts and affects all spheres of an individual’s inter-personal, social and occupational life [6]. The negative impact of depression on students continues after graduation [1] at a staggering cost [7]. It adversely affects the quality of patient care, patient safety, and professionalism. In other words, students’ emotional wellbeing is important not only for themselves but also as a factor contributing to the wellbeing of the larger society [1, 8]. Therefore, understanding how to prevent and detect depression at an early stage is a research priority in medical education. It is time to understand the factors associated with depression in school. One such factor might be the students’ study skills.

In this study, we define the term ‘study skills’ as the conscious and intentional use of processes around organizing and retaining new information, with the aim of learning more effectively [9,10]. From this perspective, the elements of study skills comprise a wide range of behaviors that students can perform before, during, and after learning to help them remember and apply the information presented in the classroom, for example the mental process of staying focused on an activity [4]. A wide body of literature exists that considers and theorizes the underlying cognitive and emotional aspects that underpin ‘learning’ (including social and emotional aspects of learning [11,12], motivation theories and self-regulated learning (SRL) theories [13, 14, 15, 16]. The current study, rather than developing or exploring the underpinning theories of learning, considers ‘study skills’ as a practical construct that assists students in their learning measurable by self-reported behaviors through an inventory.

When comparing medical students with the general population, Heinen reported that students had higher levels of perceived stress and emotional stress (depression and anxiety) [17]. However, the medical students used more self-efficacy strategies (such as positive view of obstacles and internal motivation) as personal resources which are common elements for both study skills and self-regulated learning. These personal resources acted as a buffer, but could not prevent the increased levels of perceived stress, anxiety and depression [17]. Recent findings on the relationship between self- regulated learning (SRL) and depression strongly support the view that increased use of SRL skills could help reduce depressive symptoms among students [13]. A Medline search using the key words [(Health Occupations Students OR medical students) AND Study Skills AND Depression] yielded 65 articles, but only three studies were relevant. The first was among adolescent students and studied “sluggish cognitive tempo” rather than depression [18], and the other two studies were on the impact of a self-development coaching program on medical and dental students’ psychological health and academic performance. None of these studies discussed the relationship between study skills and depression [19, 20]. A study among students of introductory psychology courses at Arkansas State University, presented in a conference 23 years ago (1992) reported that students with more depressive symptoms did not have greater difficulty with specific study behaviors [21]. However, the available version did not show the data clearly to verify the results. To the best of our knowledge, we did not come across other studies that directly compared study skills and depression. This clearly shows a gap in the literature.

A literature search on the measurement of study skills found only few validated inventories for example, LASSI (Learning and Study Strategies Inventory) [22], ASSIST (Approaches and Study Skills Inventory for Students) [23] and DCSSI tool (Denver Congos Study Scale Inventory) [24]. They have, however, several shortcomings. All inventories are impractically long. The number of questions in LASSI is 80 in ten different subscales; in ASSIST 52 in 13 different subscales; and in DCSSI 51 in six subscales. Furthermore, LASSI requires expensive subscription amounting to 4 USD per student [25]. The language of these inventories is complicated for non-native English speakers. The literature indicates that the inventory design methodologist should avoid such shortcomings [26, 27]. There are also few psychometric concerns of these inventories. For example, ASSIST reported Cronbach reliabilities ranging between 0.70 and 0.88 and somewhat lower coefficients for subscales, which ranged from 0.40 to 0.77 [28–32]. The lower coefficients resulted from the small number of items in each subscale, and that alpha values are often lower than 0.70 in these cases. Furthermore, the validity evidence pertaining to the ability of LASSI to accurately assess students’ achievement is mixed [33].

In conclusion, based on these reasons the available validated inventories had certain shortcomings. Therefore, it was decided to develop a more feasible and valid inventory, to measure purely study skills, rather than learning styles or strategies.

This study may also contribute to our understanding of depression among medical students, and whether possessing poor study skills partly have a relationship to the development of depressive symptoms. Our hypothesis was that they might be negatively related.

### Objectives

1. To validate a new instrument, Study Skill Inventory (SSI) through internal and external validation.
2. To identify the relationship between total study skills scores and depressive symptoms scores as measured by the BDI-II among medical students.

## Method

### Study design

This was a validation study of a newly developed inventory (Year 2015) followed by a cross-sectional correlational study (Year 2016).

### Target population

The target population was students of both genders attending the College of Medicine, King Saud University, Riyadh, Kingdom of Saudi Arabia.

### Characteristics of the setting

King Saud University (KSU) offers a six-year baccalaureate degree program for medicine. All students, who enroll in the medical program at KSU, must first be accepted by the unified one year program for health sciences. The criteria for acceptance in the unified program are based on the grade in the capabilities exam, accumulative exam, high schools and in the interview. All students successfully completing the curriculum of the unified program are directed to one of the four Health Faculties (Medicine, Dentistry, Pharmacy, Applied Medical Sciences) based on students’ desire, their cumulative average and the capacity of the desired Faculty. KSU operates on a single-gender basis.

### Sampling

The Open epi website was used to estimate the required sample size based on the estimated parameters for the study population namely confidence level of 95% and power of 80%. The estimated sample size was 323. With an expected non-response rate of 20%, 400 students were invited to participate.

A stratified random sampling technique with the proportional allocation method was used to determine the number of students based on sex and the year of study. Microsoft Excel program was used to generate random numbers to select the study sample within each sex and study year.

### The first validation study

#### The Study Skills Inventory (SSI) development and internal validation

Three educationists, who were expert in the field of medical education and five medical students in the third year of study reviewed the literature with the aim to develop an inventory that is relevant and easy to complete. They started with items from four study skills inventories, namely Student Success Survey & Student guide [34], Study Skills Assessment [35], Study Skills Survey [36] and Study Skills Questionnaire [37]. These inventories were not used as the main study skills tool for this research because they were not validated. The validated questionnaires such as LASSI and ASSIST were not used as a starting point because of their shortcomings as discussed earlier. Several themes discussed in the literature suggested the initial subscales (themes) for the new inventory, namely reading, concentration and memory, time management, emotional management, motivation, study methods (s), exam technique, lack of distraction and learning styles. In the final version of the SSI, only five themes were kept (S1 Table).

The first draft of the inventory consisted of 97 items/statements. Then, the educationists and students did an initial review. They were asked to mark each statement either A = important, B = borderline, C = not important at all or R = repeat/duplicate statements. The items that the respondents identified as ‘C’ or ‘R’ were deleted. The remaining items were ordered in a manner that flows in a logical sequence to make it easy to respond. The wording of all statements was kept as clear as possible. It was also decided to avoid judgmental statements and statements that contained more than one description or event.

#### Scoring system of the SSI

A four-point scoring scale was used to score each statement of the inventory:

3- The statement is always true for me (i.e. all the time = around 100% of the time).
2- The statement is usually true for me (i.e. sometimes = around 75% of the time).
1- The statement is rarely true for me (i.e. few times = around 25% of the time).
0- The statement is never true for me (i.e. almost at no times = around 0% of the time).

The 47 items were piloted on 20 students to test the logistics of data collection and clarity of the items. None of the 47 items was omitted following this pilot.

#### Factor analysis

All the 47 items were classified according to the above-mentioned themes into five subscales (factors). A confirmatory factor analysis for the five subscales was then conducted. The final inventory comprised 23 items after exclusion of all the items that had a loading of <0.4, with varimax rotation. The items fitted into their corresponding factors (subscales) and were named according to the general theme of the statements in each subscale and as per the literature reviews indicated in Table 1. The factor loadings for the 23 items and Cronbach alpha for each factor were calculated. COSMIN guideline as the framework was used as the design methodological quality (S2 Table).

**Table 1:** The loading for the 23 items and the subscales they fit.

Reading					
	TM*	C&M*	OLP*	Reading	EM*
I try to organize facts in a systematic way.	.205	.109	.132	.611	.151
I look for the main ideas as I read.	-.118	.049	.020	.636	.050
I take notes as I read my textbooks.	.295	-.048	.152	.657	.078
When reading, I highlight important passages.	.363	-.003	.093	.559	.002
I see learning as something I will be doing all throughout my life. (reading)	-.066	.210	.054	.490	.279
**Concentration and Memory**					
I study even when less important things distract me.	-.039	.638	.107	.006	.116
I give full attention to the tasks.	-.045	.578	.016	.112	.307
I keep up with assignments, readings and tests preparations, avoiding any delays.	.394	.566	.100	.034	.187
I keep study time a priority, saying “no” to social demands and extracurricular events.	.302	.653	.123	.046	-.172
I avoid activities, which tend to interfere with my planned schedule.	.322	.635	.084	.090	-.051
**Time Management**					
At the beginning of the term, I make up my activity and study schedules.	.743	.076	.064	.088	.055
I break assignments into manageable parts.	.696	.119	.041	.069	.129
I use a “to do” list to keep track of the tasks.	.723	.149	.025	.080	.007
I plan my day by deciding what is important to do.	.520	.451	.091	.082	.234
I outline specific goals for my study time.	.397	.352	.076	.313	.111
**Emotional Management**					
I plan regular times for fun.	.160	.116	.006	-.015	.690
I take relaxation or rest time when under stress.	-.044	-.024	.023	.137	.733
I regularly try to motivate myself to keep up with the planned schedule.	.332	.113	.088	.246	.574
I try to get rid of negative thoughts and worrying while studying.	.046	.159	.253	.186	.556
**Other learning practices**					
When I do not understand something, I get help from classmates.	.188	-.009	.520	-.235	.282
I volunteer answering to questions posed by instructors in the class.	.033	.134	.805	.121	.013
I participate in class discussions.	.008	.122	.823	.153	.002
I ask the instructor questions when clarification is needed.	.065	.107	.662	.233	.103

TM*: Time management; C&M*: Concentration and memory; OLP*: Other learning practices; EM*: Emotional management

The results presented in the rest of the paper are from the final version of the inventory (23 items) under the following categories:

- Reading texts: 5 items = 15 (maximum score)
- Concentration and memory: 5 items = 15 (maximum score)
- Time management: 5 items = 15 (maximum score)
- Emotional management: 4 items = 12 (maximum score)
- Other learning practices: 4 items = 12 (maximum score)

#### External validation of the SSI

Eighty-four volunteer medical students completed the SSI and the ASSIST simultaneously to assess the external validity of the SSI. The ASSIST was used here only for the external validation. Despite the limitations of the ASSIST, it was used for external validation as one of the best available options.

### The second correlational study

Instruments used in this study were; BDI-II, SSI and a demographic questionnaire (which collected participants’ age, sex, GPA, marital status and study year).

#### Beck Depression Inventory II (BDI-II)

It is a psychometric instrument that measures depressive symptoms, with broad applicability worldwide in both research and clinical practice [38,39]. It is a 21-item self-report instrument for measuring the severity of depression. Each answer is scored on a scale of 0 to 3 for each item [40]. The total maximum score for all items thus is 63 [41]. A score of 0–13 is considered minimal, 14–19 mild, 20–28 moderate, and 29–63 severe depression; and scores above 13 are considered significant depressive symptoms [40]. BDI-II was selected because of its wide use, specificity and sensitivity in detecting depression among university students, in addition to its high reliability, and concurrent and content validity [38].

#### Data collection

BDI-II, the SSI and the demographic questionnaire were administered at the same time and were uploaded to Survey Monkey through a link sent to the selected students. In survey Monkey, if respondents do not answer a required question, they will not be able to advance to the next page until they answer the said question. Therefore, there were no missing items. The first page of the online inventory explained the aims of the study and assured anonymity and confidentiality. The participation was on a voluntary basis.

#### Data analysis plan

The Statistical Package for the Social Sciences (SPSS) [version 21] was used for data analysis. Descriptive statistics such as frequency, percentage, mean, and standard deviation were calculated for socio-demographic data. One-way analysis of variance (ANOVA) was used to compare the mean values of quantitative variables with Tukey and Scheffe Test as the multiple comparison post-hoc test. Pearson correlation coefficient was computed to quantify the linear relationship between SSI total score and BDI total score of each participant. All statistical comparisons were considered significant at a level of less than 0.05.

### Ethics approval and consent to participate

Informed consent was obtained from all students prior to data collection. All the selected respondents were given assurance of confidentiality that the information gathered will be used exclusively for research purposes. The current study was approved by the Institutional Review Board of the College of Medicine; KSU (reference no F24 dated 16/12/2015).

## Results

### Participants

The total number of students who filled out the SSI and the BDI II were 372 and 328 (response rate of 93% and 82%) respectively. Male students (57.0%) outnumbered their female counterparts. The mean age of the students was 21.30 ± 1.47 years. Most of the participants were single (98.0%) (Table 2).

**Table 2:** Distribution of socio-demographic characteristics of study subjects (n = 328).

Socio-demographic characteristics		Number (%) of students
**Sex**	Male	187 (57.0)
	Female	141 (43.0)
**Marital status**	Single	321 (97.9)
	Married	5 (1.5)
	Divorced	2 (0.6)
**Study year**	First year	72 (22.0)
	Second year	59 (18.0)
	Third year	72 (22.0)
	Fourth year	58 (17.7)
	Fifth year	67 (20.4)
**Age**	**≤ 22**	246 (75.0)
	**> 22**	82 (25.0)

#### Factor statistics

The loading for the 23 items and the subscales they fit are shown (Table 1). The items fit into five factors and with an Eigen value >1, they explained a cumulative variance of 52%. The only item that produced mixed loadings was ‘I outline specific goals for my study time’. However, this item was included, as the highest loading that this item produced marginally met the criteria for inclusion; i.e. a factor loading of 0.4.

The mean for the total SSI score was 43.9 which is 64% of the maximum score of 69. For the five factors (domains), students had the highest mean score on the time management domain (95% of the maximum score) and the lowest score on the concentration (32%) and reading domains (57%) (Table 3).

**Table 3:** The SSI inventory total and five factors statistics for the study sample.

	Score range	Mean	SD	Mean % of the total	Cronbach’s Alpha reliability	Cronbach’s Alpha if Item Deleted
**Reading**	0–15	8.6	2.2	57	.652	.73
**Time management**	0–15	14.3	4.5	95	.755	.65
**Emotional management**	0–15	8.8	2.2	59	.661	.73
**Concentration and memory**	0–12	3.8	1.1	32	.697	.76
**Other learning practices**	0–12	8.7	2.7	72	.708	.72
**Total**	0–69	43.9	8.8	63.1	0.84	

The Cronbach alpha reliability for the total SSI was 0.84 and for the subscales, alpha reliability ranged from 0.65 to 0.76 (Table 3 and S1 Data).

#### External validation

The result of the external validation showed a significant positive correlation (r = 0.511, p<0.0001) between the scores of SSI and ASSIST inventories. It was observed by the author (MA) who distributed the inventories that the respondents took longer time to complete ASSIST compared to the SSI. Some of the ASSIST items had many queries for clarification by the respondents.

#### Correlation between SSI and BDI-II

There was a statistically significant, low-moderate, negative correlation between the BDI-II score and the SSI total score (r = -0.348, p< 0.0001). Similarly, a statistically significant difference was found between the mean SSI total scores of respondents belonging to the four categories of depressive symptoms (F ratio = 13.303; p< 0.0001). (Table 4 and S2 Data).

**Table 4:** Comparison of the respondents’ means of SSI total scores with different severity levels of depression.

Depression categories	Number of students (%)	Total Study skills score Mean (+SD)	F-value (p-value)	95% Confidence Interval for Mean		Tukey and Scheffe Multiple comparison test			
				Lower Bound	Upper Bound	minimal	mild	moderate	severe depression
Minimal (normal)	236 (72)	45.61 (8.07)	13.303 <0.0001	44.5712	46.6407	1.00	.012	.000	.002
Mild depression	52 (16)	41.37 (8.74)		38.9314	43.7993		1.00	.434	.194
Moderate depression	30 (9)	38.20 (9.37)		34.7021	41.6979			1.00	0.792
Severe depression	10 (3)	35.10 (9.09)		28.6007	41.5993				1.00
Total	328	43.94 (8.79)		42.9814	44.8906				

## Discussion

The analysis of the newly developed SSI data produced results that support its reliability and validity. The SSI total score had a significant negative association with the BDI-II depression score, in other words, poor study skills were correlated with higher depressive symptoms.

The fitting of the 23 items into their corresponding themes supported the instrument validity. Furthermore, although SSI inventory was relatively short, it had acceptable reliability.

When comparing the SSI with the LASSI, it was observed that two of the 10 different subscales of LASSI perfectly matched the SSI domains of Concentration (CON), and Time Management (TMT). Similarly, Reading (5 items) in the SSI was close to the Selecting Main Idea (SMI) domain in LASSI. SSI had other items (not included in LASSI) to address certain study skills, e.g. taking notes. Furthermore, two domains in LASSI (anxiety and motivation) are related to depression which is the factor being tested in the current study for correlation with study skills. However, these are best measured using more specific tools for these attributes, rather than study skills inventories.

Reading and Concentration & memory domains in the SSI in the current study had lower scores as compared to the other three domains. These two domains are relevant to independent learning. This is consistent with what Frambach et al. [42] reported regarding context and culture, that unlike Asian and Western students, their Arab counterparts needed more guidance even in a student-centered strategy like problem-based learning (PBL).

There is a scarcity of studies that directly investigated the relationship between the students’ total SSI scores and depressive symptoms. The current study finding of a significant inverse correlation between the students’ total SSI scores and the BDI-II scores is a further step to better understand student depression phenomenon. Overall, the findings from the current study shed light on the hither-to unexplored relationship between depressive symptoms and study skills among health college students (in particular) and university students in general. It adds the study skills as possible factor that needs to be confirmed in future studies.

Shahidi et al. [43] suggested that less than desirable study skills may lead to mental slowness, stress and anxiety. In other words, the use of better skills may facilitate studying, making it faster and more pleasant and thereby reducing the students’ boredom and emotional cost. Similarly, Bandura et al. [44] suggested that social and academic inefficacy contributes to concurrent and subsequent depression both directly and through their impact on academic achievement. Therefore, in line with this theory, the current study found an association between ineffective (poor) study skills and higher depressive symptoms. In contrast, Tobias [45] observed the ineffective study habits or low study skills of the anxious students during the time of tests (exams). He suggested that test anxiety reduces the performance through reduction of the cognitive capacity available for task solution [44]. This theory related poor study skills with anxiety but not with depressive symptoms. As the current study is a correlational study, causality in either direction (depressive symptoms causing poor study skills, or poor study skills leading to depressive symptoms) cannot be claimed.

### Limitations

As stated earlier, this is an initial correlational study and cannot claim causality in either direction. It can well be that depressive symptoms interfered with the student’s reading ability or memory and concentration which resulted in poor study skills. The study was conducted on students of a single medical school, which may reduce the generalizability of the findings. Another limitation is the uncertainty of respondents’ ability to accurately respond to each statement; i.e. difficulties related to self-assessment.

## Conclusion

This study showed evidence for acceptable reliability and validity of the newly developed SSI. The results of the study suggest that poor study skills correlate with depressive symptoms.

## Recommendations

Further studies are needed to confirm the current study findings and to elucidate whether poor study skills and habits contribute to higher depressive symptoms directly or through lower performance in exams, or whether depressive symptoms lead to poor study habits such as surface learning. Therefore, it is recommended to design two types of studies before any firm conclusions and recommendations can be made. The first is a controlled trial among two groups of depressed students to assess the impact of a study skills program on the depressive symptoms of the index group; i.e. the effect of adding a study skills course to the curriculum needs investigation. The second is to follow up a group of medical students who have high depressive symptoms and to find out if the treatment of depression improves their study skills.

## Supporting information

S1 Table The Study skills inventory. (DOCX)

S2 Table COSMIN guidelines as the framework for the design methodological quality. (DOCX)

S1 Data The reliability analysis output for the five factors. (XLS)

S2 Data Output of the correlation between the mean study skills scores and depression categories. (XLS)

## Abbreviations

BDI II: Beck Depression Inventory II
SSI: Study Skills Inventory
