# Supplementary material for: Validation of a new study skills scale to provide an explanation for depressive symptoms among medical students
Source: PLoS One. 2018 Jun 25;13(6):e0199037. doi: 10.1371/journal.pone.0199037 (PMC6016898; doi:10.1371/journal.pone.0199037)
Supplement: S1 Table — (DOCX) [file pone.0199037.s001.docx]

**The Study Skills Inventory**

|  | **Reading text** | Always | Usually | Rarely | Never |
| --- | --- | --- | --- | --- | --- |
|  | I try to organize facts in a systematic way. |  |  |  |  |
|  | I look for the main ideas as I read. |  |  |  |  |
|  | I take notes as I read my textbooks. |  |  |  |  |
|  | When reading, I highlight important passages. |  |  |  |  |
|  | **Concentration and memory** | Always | Usually | Rarely | Never |
|  | I study even when less important things distract me |  |  |  |  |
|  | I give full attention to the tasks. |  |  |  |  |
|  | I keep up with assignments, readings and tests preparations, avoiding any delays |  |  |  |  |
|  | I keep study time a priority, saying “no” to social demands and extracurricular events |  |  |  |  |
|  | I avoid activities, which tend to interfere with my planned schedule. |  |  |  |  |
|  | Time management | Always | Usually | Rarely | Never |
|  | At the beginning of the term, I make up my activity and study schedules |  |  |  |  |
|  | I break assignments into manageable parts. |  |  |  |  |
|  | I use a “to do” list to keep track of the tasks. |  |  |  |  |
|  | I plan my day by deciding what is important to do |  |  |  |  |
|  | I outline specific goals for my study time. |  |  |  |  |
|  | **Emotional management** | Always | Usually | Rarely | Never |
|  | I plan regular times for fun. |  |  |  |  |
|  | I take relaxation or rest time when under stress. |  |  |  |  |
|  | I regularly try to motivate myself to keep up with the planned schedule |  |  |  |  |
|  | I try to get rid of negative thoughts and worrying while studying |  |  |  |  |
|  | **Other learning practices** | Always | Usually | Rarely | Never |
|  | When I do not understand something, I get help from classmates |  |  |  |  |
|  | I participate in class discussions. |  |  |  |  |
|  | I volunteer answers to questions posed by instructors in the class. |  |  |  |  |
|  | I see learning as something I will be doing all throughout my life. |  |  |  |  |
|  | I ask the instructor questions when clarification is needed. |  |  |  |  |
