# Supplementary material for: Validation of a new study skills scale to provide an explanation for depressive symptoms among medical students
Source: PLoS One. 2018 Jun 25;13(6):e0199037. doi: 10.1371/journal.pone.0199037 (PMC6016898; doi:10.1371/journal.pone.0199037)
Supplement: S2 Table — (DOCX) [file pone.0199037.s002.docx]

COSMIN guideline as the framework for the general design methodological quality

| Box A. Internal consistency | yes | no | NA |
| --- | --- | --- | --- |
| 1 Does the scale consist of effect indicators, i.e. is it based on a reflective model? | √ |  |  |
| Design requirements | Yes | no | NA |
| 2 Was the percentage of missing items given? | √ |  |  |
| 3 Was there a description of how missing items were handled? | √ |  |  |
| 4 Was the sample size included in the internal consistency analysis adequate? | √ |  |  |
| 5 Was the uni dimensionality of the scale checked? i.e. was factor analysis or IRT model applied? | √ |  |  |
| 6 Was an internal consistency statistic calculated for each (unidimensional)  (sub) scale separately? | √ |  |  |
| 7 Were there any important flaws in the design or methods of the study? |  | √ |  |
| Statistical methods | yes | no | NA |
| 8 for Classical Test Theory (CTT): Was Cronbach’s alpha calculated? | √ |  |  |
| 9 for dichotomous scores: Was Cronbach’s alpha or KR-20 calculated? |  |  | √ |
| 10 for IRT: Was a goodness of fit statistic at a global level calculated? e.g. χ2, reliability coefficient of estimated latent trait value (index of (subject or item) separation) |  |  | √ |

| Box B. Reliability: relative measures (including test-retest reliability, inter-rater reliability and intra-rater reliability) |  | | |  |  |
| --- | --- | --- | --- | --- | --- |
| Design requirements | Yes | | | no | NA |
| 11 Were at least two measurements available? |  | | |  | √ |
| 12 Were the administrations independent? |  | | |  | √ |
| 13 Was the time interval stated? |  | | |  | √ |
| 14 Were patients stable in the interim period on the construct to be measured? |  | | |  | √ |
| 15 Was the time interval appropriate? |  | | |  | √ |
| 16 Were the test conditions similar for both measurements? e.g. type of administration, environment, instructions |  | | |  | √ |
| 17 for continuous scores: Was an intraclass correlation coefficient (ICC) calculated? |  | | |  | √ |
| 18 for dichotomous/nominal/ordinal scores: Was kappa calculated? |  | |  | | √ |
| 19 for ordinal scores: Was a weighted kappa calculated? |  | |  | | √ |
| 20 for ordinal scores: Was the weighting scheme described? e.g. linear, quadratic |  |  | | | √ |

| Box C. Content validity (including face validity) |  |  |  |
| --- | --- | --- | --- |
| General requirements | Yes | no | NA |
| 21 Was there an assessment of whether all items refer to relevant aspects of the  Construct to be measured? | √ |  |  |
| 22 Was there an assessment of whether all items are relevant for the study  Population? (e.g. age, gender, disease characteristics, country, setting) | √ |  |  |
| 23 Was there an assessment of whether all items are relevant for the purpose of the measurement instrument? (discriminative, evaluative, and/or predictive) | √ |  |  |
| 24 Was there an assessment of whether all items together comprehensively reflect the construct to be measured? | √ |  |  |

| Box D. Structural validity |  |  |  |
| --- | --- | --- | --- |
| Statistical methods | Yes | no | NA |
| 25 for CTT: Was exploratory or confirmatory factor analysis performed? | √ |  |  |
| 26 for IRT: Were IRT tests for determining the (uni-) dimensionality of the items  Performed? | √ |  |  |
